# Modeling of Laser Beam Absorption in a Polymer Powder Bed

**DOI:** 10.3390/polym10070784

**Published:** 2018-07-17

**Authors:** Fuad Osmanlic, Katrin Wudy, Tobias Laumer, Michael Schmidt, Dietmar Drummer, Carolin Körner

**Affiliations:** 1 Joint Institute of Advanced Materials and Processes, Friedrich-Alexander-Universität Erlangen-Nürnberg Dr.-Mack-Str. 81, 90762 Fuerth, Germany; carolin.koerner@fau.de; 2 Collaborative Research Center 814-Additive Manufacturing, Friedrich-Alexander-Universität Erlangen-Nürnberg, Am Weichselgarten 9, 91058 Erlangen, Germany; wudy@lkt.uni-erlangen.de (K.W.); Tobias.Laumer@continental-corporation.com (T.L.); michael.schmidt@lpt.uni-erlangen.de (M.S.); drummer@lkt.uni-erlangen.de (D.D.); 3 Institute of Polymer Technology, Friedrich-Alexander-Universität Erlangen-Nürnberg, Am Weichselgarten 9, 91058 Erlangen, Germany; 4 BLZ Bayerisches Laserzentrum GmbH, 91052 Erlangen, Germany; 5 Chair of Materials Science and Engineering for Metals, Friedrich-Alexander-Universität Erlangen-Nürnberg, Martensstr. 5, 91058 Erlangen, Germany

**Keywords:** additive manufacturing, laser absorption, powder bed, polyamide 12, PA12

## Abstract

In order to understand the absorption characteristic, a ray trace model is developed by taking into account the reflection, absorption and refraction. The ray paths are resolved on a sub-powder grid. For validation, the simulation results are compared to analytic solutions of the irradiation of the laser beam onto a plain surface. In addition, the absorptance, reflectance and transmittance of PA12 powder layers measured by an integration sphere setup are compared with the numerical results of our model. It is shown that the effective penetration depth can be lower than the penetration depth in bulk material for polymer powders and, therefore, can increase the energy density at the powder bed surface. The implications for modeling of the selective laser sintering (SLS) process and the processability of fine powder distributions and high powder bed densities are discussed.

## 1. Introduction

Selective laser sintering of polymers (SLS) is a powder- and laser-based additive manufacturing technique. Due to its thermal stability, build accuracy and mechanical properties, selective laser sintering of plastics shows an excellent capability to manufacture serial components [1,2]. The lack of fundamental process understanding in selective laser sintering leads to process interruptions and to defective parts, which hampers the breakthrough of selective laser sintering in many industrial sectors. Experimental analysis of the beam-powder interaction in combination with modeling could fundamentally enhance the understanding of SLS processing conditions [3].

During SLS, a CO${}_{2}$ laser selectively melts the thermoplastic powder layer by layer. Exposed to laser irradiation, the plastic material is transformed from an entropy elastic state to a viscous state. In contrast to selective laser melting of metals and according to the model for quasi-isothermal laser sintering, the molten thermoplastic materials remain in the viscous state during the whole laser sintering process. The temperature-dependent viscosity and surface tension determine the viscous flow of the thermoplastic melt. The actual temperature and thus the viscosity are influenced by the absorption behavior of the powder mainly. The resulting material and mechanical properties are dependent on the laser energy input which is dominated by the interaction of the beam with the powder bed [4,5]. While thermal modeling of the process is conducted with simplified absorption models [6,7], a mesoscopic model at the powder scale is still missing. Therefore, in this work, a ray tracing absorption model for thermoplastic powders is developed and validated with theoretical and experimental data.

## 2. Absorption Model

The basic idea of the absorption model is to trace the path of photons (ray), emitted by a light source (e.g., a laser) by considering the reflection and refraction at the interface of particles and the atmosphere, while absorbing the energy according to the Beer–Lambert law (Equation (1)) along its path in dense media. The media, e.g., within a powder particle, are assumed to be linear. The radiation intensity distribution *I* in a medium for a single wavelength is given by:

(1) $$\begin{array}{c} I\left(x\right)=I_{0}\;e^{-\mu x} \end{array}$$

where *x* is the path, μ is the attenuation coefficient for a unique wavelength in a given medium and $I_{0}$ is the initial intensity. To calculate the path of light, a ray is cast at the coordinates of its source ${\vec{x}}_{0}$ with the propagation vector $\vec{k}$ normalized to unit length and the energy $E_{0}$. The propagation is discretized into equal steps *n* of length Δl, and at each position ${\vec{x}}_{n}$, the medium is sampled and the energy absorbed ΔE by the medium calculated.

(2) $$\begin{array}{cc} {\vec{x}}_{n} & ={\vec{x}}_{0}+n\;\Delta l\;\vec{k} \end{array}$$

(3) $$\begin{array}{cc} \Delta E\left({\vec{x}}_{n}\right) & =E\left({\vec{x}}_{n-1}\right)\left(1-e^{-\mu \Delta l}\right) \end{array}$$

At each step, the remaining Energy $E_{n}$ of the beam needs to be updated.

(4) $$\begin{array}{c} E\left({\vec{x}}_{n}\right)=E\left({\vec{x}}_{n-1}\right)-\Delta E\left({\vec{x}}_{n}\right) \end{array}$$

**Figure 1:**
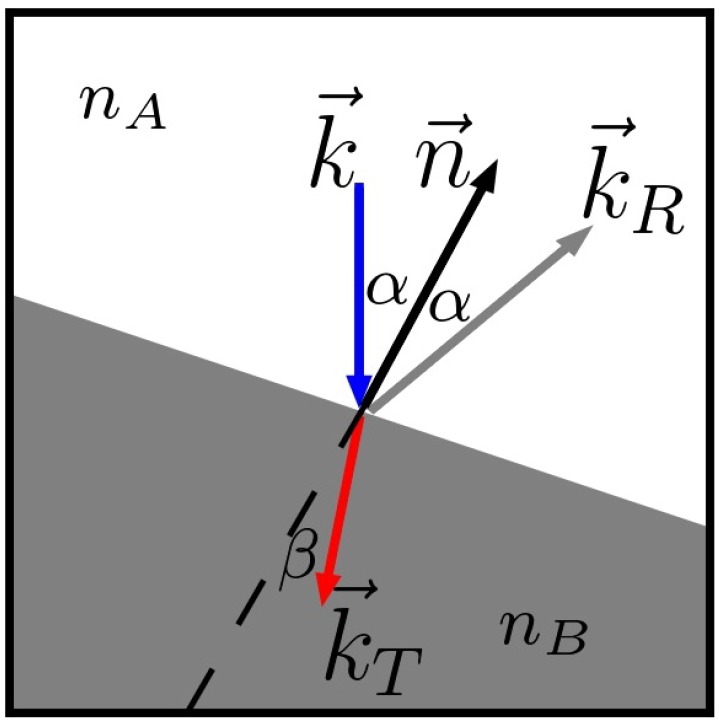
The incoming ray $\vec{k}$ is divided into a reflected ${\vec{k}}_{R}$ and refracted ${\vec{k}}_{T}$ part at the interface of media *A* and *B*. α and β are the reflection and refraction angle, respectively.

The ray propagates until it encounters a change in media. At that point, the initial ray is terminated, and two new rays are cast (Figure 1), accounting for reflection and refraction at the interface of two different media.

The reflection angle α has the same magnitude as the angle of the incoming ray with respect to the surface normal $\vec{n}$.

(5) $$\begin{array}{c} \alpha =\cos^{-1}\left(-\vec{k}\cdot \vec{n}\right) \end{array}$$

For the refraction angle β, Snell’s law [8] can be applied:

(6) $$\begin{array}{c} \sin\left(\beta \right)=\frac{n_{A}}{n_{B}}\;\sin\left(\alpha \right) \end{array}$$

with $n_{A}$ being the refractive index of the medium *A*. The direction of the reflected ${\vec{k}}_{R}$ and refracted ${\vec{k}}_{T}$ ray can be easily calculated by multiplying the normal vector by the rotation matrix using the corresponding angle. The remaining energy of the incoming ray $E_{I}$ is divided between the two new rays. By using the Fresnel equations for unpolarized light and non-magnetic media, the energy portion for the reflected ray $E_{R}$ can be calculated as:

(7) $$\begin{array}{cc} E_{R} & =R\;E_{I} \end{array}$$

(8) $$\begin{array}{cc} R & =\frac{1}{2}\left(\;{\left|\frac{n_{A}\cos\alpha -n_{B}\cos\beta }{n_{A}\cos\alpha +n_{B}\cos\beta }\right|}^{2}+{\left|\frac{n_{A}\cos\beta -n_{B}\cos\alpha }{n_{A}\cos\beta +n_{B}\cos\alpha }\right|}^{2}\right) \end{array}$$

The energy for the transmitted ray $E_{T}$ is given by:

(9) $$\begin{array}{cc} E_{T} & =\left(1-R\right)\;E_{I} \end{array}$$

By taking into account the starting point, the direction vector and the energy, new rays are cast and propagated analogously to the initial ray.

## 3. Implementation

**Figure 2:**
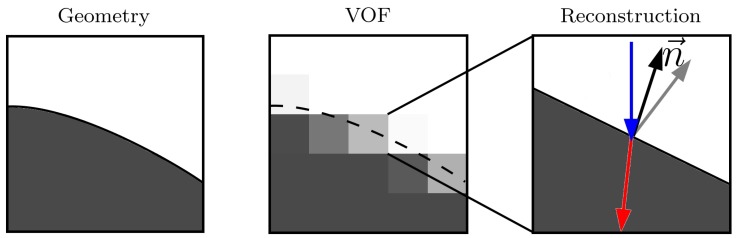
(**Left**) Arbitrary geometry *G*. (**Middle**) Volume of fluid (VOF) representation of *G* on a 5 × 5 grid. The exact surface is indicated by the dashed line. The grey scale shows the volume fraction of the geometry within one cell. (**Right**) Reconstruction of the surface in one cell.

The absorption model described in the previous section can be applied to any simulation method that provides the necessary geometrical information. However, this work will focus on the implementation within the volume of fluid method (VOF) in 2D. It is straight forward to extend the scheme to three dimensions. The VOF in combination with the lattice Boltzmann method has been proven to be very effective when it comes to simulating complex geometries like a powder bed in additive manufacturing [9,10,11]. To represent a given geometry, the space in the simulation domain is discretized into cells. According to its occupation level by that geometry, each cell gets a volume fraction φ between zero and one (Figure 2).

In general, the information of the exact shape of the geometry is lost after the initialization, but the big advantage is that the surface can be locally reconstructed up to certain accuracy; therefore, the algorithm is ideal for parallel computing. The absorption model requires the surface normal $\vec{n}$ to calculate ${\vec{k}}_{R}$ and ${\vec{k}}_{T}$. One way to estimate the normal vector within one cell at its center $\vec{x}$ is to sum over the displacements vectors ${\vec{x}}_{i,j}$ of the neighboring cells weighted by their inverse volume fraction [12].

(10) $$\begin{array}{cc} {\vec{n}}^{*}\left(\vec{x}\right) & =\sum_{i,j=-2}^{2}\left(1-\varphi \left(\vec{x}+{\vec{x}}_{i,j}\right)\right)\;{\vec{x}}_{i,j} \end{array}$$

(11) $$\begin{array}{cc} \vec{n} & =\frac{{\vec{n}}^{*}}{\left|{\vec{n}}^{*}\right|} \end{array}$$

(12) $$\begin{array}{cc} {\vec{x}}_{i,j} & =i\;{\vec{e}}_{x}+j\;{\vec{e}}_{y} \end{array}$$

where ${\vec{e}}_{x}$ and ${\vec{e}}_{y}$ are the unit vector in Cartesian coordinates. A linear reconstruction of the surface within the cell can be calculated with a template sphere method [12]. Due to the simplicity and to save computational time, the surface in this work is defined when a ray propagates from a cell with φ=0 to a cell with φ>0 and vice versa.

## 4. Validation

The developed model is validated with an analytical solution for the irradiation of a laser beam onto a plain surface and versus experimental data on the absorptance, reflectance and transmittance of PA12 powder layers.

### 4.1. Plain Surface

A simple setup to validate the absorption model with an analytic solution is the irradiation of a laser beam onto a plain surface with different incident angles. The laser power $P\left(x\right)$ distribution is assumed to be Gaussian,

(13) $$\begin{array}{c} P\left(x\right)=P_{0}\;\frac{1}{\sigma \sqrt{2\pi }}e^{-\frac{1}{2}{\left(\frac{x-x_{0}}{\sigma }\right)}^{2}} \end{array}$$

with $P_{0}$ being the beam power, $x_{0}$ the center of the beam and the value of the beam diameter being 4σ=400μm, which is a common diameter in SLS [7,13,14]. Each ray is initialized at the top of the simulation domain in the middle of a cell at the position $x_{B}$ and $y_{B}$. The direction of the propagation vector is calculated by using the given incident angle. The energy of each ray is estimated by sampling the power distribution and multiplying with the special resolutions Δx and time discretization Δt:

(14) $$\begin{array}{c} E\left(x_{B}\right)=P\left(x_{B}\right)\cdot \Delta x\cdot \Delta t \end{array}$$

**Figure 3:**
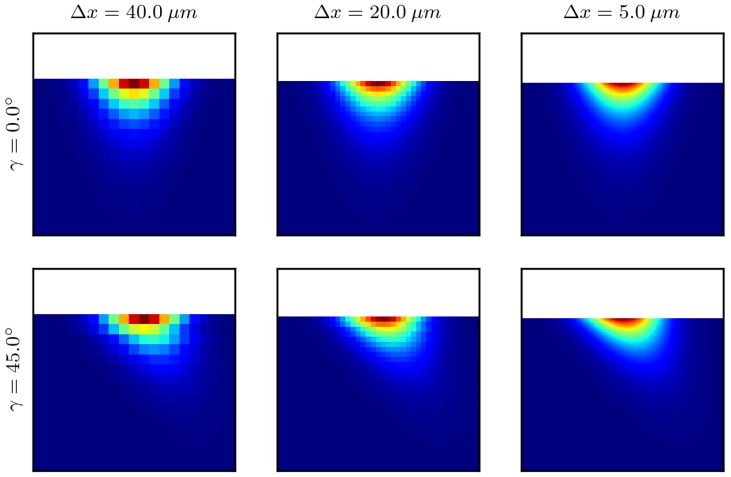
Relative intensity distribution of a laser irradiating onto a flat surface with two incident angles γ of $0^{\circ }$ (upper row) and $45^{\circ }$ (lower row) for three different spatial resolutions $\Delta x=\left[40\;\mu m,\;20\;\mu m,\;5\;\mu m\right]$ (left to right).

Figure 3 shows the relative intensity distribution into a flat surface with an incident angle γ of $0^{\circ }$ and $45^{\circ }$ for three different values of spatialresolutions. The attenuation coefficient is set to $\mu =10^{-2}\;\frac{1}{\mu m}$, and the step length of the ray tracer is Δl=0.01Δx. The incoming beam is initialized with one ray per cell. A qualitative good agreement is found with the expected distribution given by the Beer–Lambert law (Equation (15)), in that the intensity declines exponentially along the propagation direction. Furthermore, the power distribution perpendicular to the propagation direction follows the Gaussian distribution given by Equation (14).

**Figure 4:**
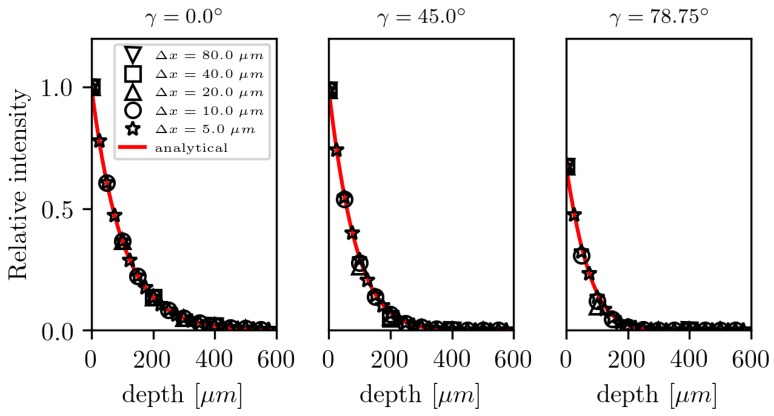
Comparison of the analytical (Equation (15)) and numerical relative intensity distribution in the beam center along the normal direction for five resolutions and three incident angles.

Figure 4 compares the relative intensity distribution in the beam center along the normal direction for five resolutions and three incident angles with its analytical solution. The radiation intensity distribution in a half plan can be calculated as a product of the given power distribution in the x-direction (Equation (14)) with the Beer–Lambert law (Equation (1)) along its refraction direction.

(15) $$\begin{array}{c} I\left(x,y,\beta \right)=\frac{P_{0}}{\sigma \sqrt{2\pi }}e^{-\frac{1}{2}{\left(\frac{x^{*}-x_{0}}{\sigma }\right)}^{2}}\;e^{-\mu y^{*}} \end{array}$$

To account for refraction, a projection of the distribution is applied, which is given by:

(16) $$\begin{array}{cc} x^{*} & =x-\sqrt{{y^{*}}^{2}-y^{2}} \end{array}$$

(17) $$\begin{array}{cc} y^{*} & =\frac{y}{\cos\beta } \end{array}$$

with β being calculated by Snell’s law (Equation (6)) using the given incident angle γ and the refractive index of the medium. Very good agreement is found for all tested cases.

### 4.2. Polyamide 12 Powder Bed

**Figure 5:**
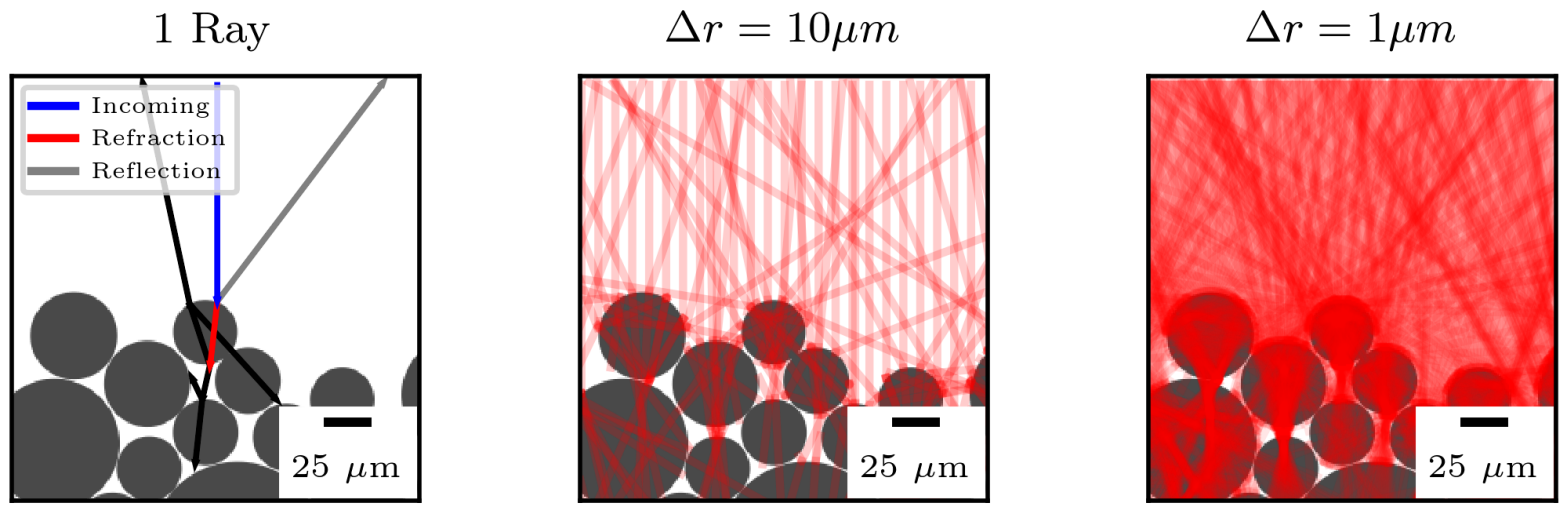
Trace of a ray cast from the top of the simulation domain propagating towards a powder bed. From left to right, the number of rays is increased.

The second test case is the absorption of a laser in a PA12 powder bed. Figure 5 (left) illustrates the trace of one single ray cast from the top of the simulation domain propagating towards a powder particle. At the intersection of the interface and the incoming ray, two new rays are cast. The reflected ray points away from the powder bed and propagates until it reaches the boundary of the simulation domain. The refracted ray transverses the powder particle and deposits its energy according to Equation (3). At the intersection, new rays are cast accounting for reflection and refraction. The traces of two different spacings for the casting distance Δr are shown in Figure 5 (middle and right). The refractive index of the atmosphere is set to $n_{atm}=1$, while the refractive index of the medium is $n_{med}>1$. Therefore, the ray is refracted towards the normal vector of the surface. Round powder particles will focus the beam and create hot spots in the powder bed.

**Figure 6:**
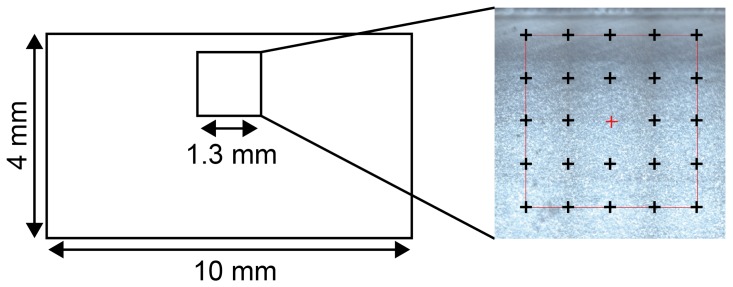
Sampling area for infrared spectroscopy mapping and positions for infrared spectra.

To determine the attenuation coefficient of polyamide 12 (PA12), films with different thicknesses were prepared. Injection-molded PA12 (material: PA2200, EOS GmbH) tensile test bars were used for sample preparation. Before injection molding, the powder raw material has passed through a compounder to generate granules, which can be injection molded. To produce thin layers with a defined thickness, a microtome from Leica Reichert Jung Type Polycut E was used. The film thickness varies between 3 and 81μm. The specimen thickness was measured with a measuring gauge. Afterward, the thin films was placed under the FTIR microscope Type Nicolet Continuum Infrared Microscope (Thermo Scientific, Germany, Langenselbold). An objective lens with a magnification of 15 was used, and the images were taken under polarized light. Before each measurement, a background measurement was taken, which was subtracted from the raw data of each measurement. Infrared spectra between 400 and 4000 cm${}^{-1}$ were taken on 25 positions over the cross-section via microscopic mapping (Figure 6).

**Figure 7:**
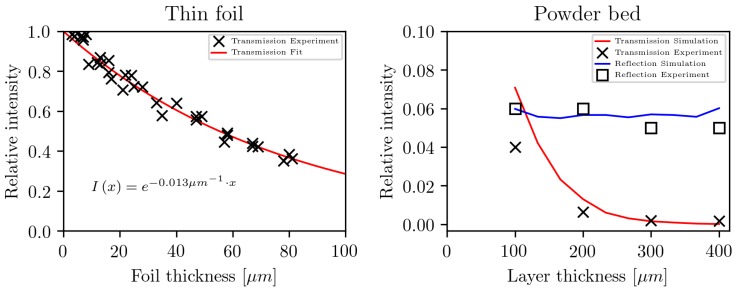
(**Left**) Relative transmission in a thin foil of PA12 with different film thicknesses. (**Right**) Comparison of the relative transmission and reflection in a PA12 powder bed between experimental data [15] and the simulation.

For the analysis, an infrared spectrometer Nicolet 6700 (Thermo Scientific, Germany, Langenselbold) was used. For each spectra, the absorption at a wavelength of 10.6μm (or wavenumber of 943 cm${}^{-1}$) was evaluated. This wavelength is of main interest due to the use of a CO${}_{2}$ laser in selective laser sintering. To analyze the absorption at 10.6μm depending on the film thickness, 33 films with different thicknesses were analyzed. For statistical reasons, the average value of the 25 measurement positions for one film was built. The measured relative transmission is shown in Figure 7 together with a fit to the Beer–Lambert law.

Figure 7 (right) shows the comparison of the relative transmission and refection in a PA12 powder bed between experimental data [15] and the simulation, using the presented absorption model. The refractive index of the medium $n_{med}=1.7$ is chosen so that the simulation results reproduce the experimental values, which are independent of the layer thickness, and the attenuation coefficient is set to $\mu =0.013\frac{1}{\mu m}$, as determined by the experiment in the thin foil. The calculated values are in good agreement with the experimental findings, while a slight overestimation of the transmission in the simulation results can be observed. This may be caused due to the neglect of diffusive scattering and the assumption of perfect spherical particles with no impurities.

## 5. Penetration Depth in a Powder Bed

A uniform radiation source is chosen to study the absorption characteristics in a powder bed. The relative density $\rho_{rel}$ and the refractive index of the medium are varied. The penetration depth λ, which is defined as the inverse of the attenuation coefficient, is set to $\lambda_{0}=100\;\mu$m. The powder diameter distribution density *q* is given by fitting a log-normal distribution:

(18) $$\begin{array}{c} q\left(\bar{d}\right)=\frac{1}{\sqrt{2\pi }\sigma \bar{d}}\;\exp\left(-\frac{{\left(\ln\left(\bar{d}\right)-\mu_{d}\right)}^{2}}{2\sigma^{2}}\right) \end{array}$$

to the normalized data given by Drummer et al. [16], with a median particle size of $d_{3,50}=\;60\;\mu$m and the parameters σ=0.25 and $\mu_{d}=4.1$. The correlation with the median particle size is given by:

(19) $$\begin{array}{c} {\bar{d}}_{3,50}=\exp\left(\mu_{d}\right) \end{array}$$

**Figure 8:**
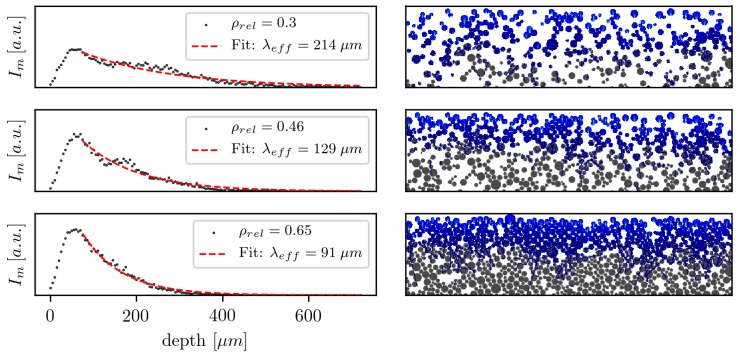
(**Right**) Three powder beds with their relative absorbed intensity distributions. (**Left**) The mean intensity $I_{m}$ over the powder bed depth for the corresponding relative densities with $n_{med}=1.7$ and $\lambda_{0}=100\;\mu$m.

The dimensionless particle diameter $\bar{d}$ is normalized to the μm scale. The powder bed is generated by randomly sampling a diameter between 10μm and 150μm. The diameter is accepted with the probability $q\left(\bar{d}\right)$ and placed into the simulation domain by randomly initializing the particle at the top of the domain and minimizing its potential energy. The algorithm is repeated until the desired fill level is reached. Finally, powder particles are randomly removed to adjust the relative density. Three resulting powder beds with the their intensity distributions are shown in Figure 8. Commercial PA12 powder (type PA2200, EOS GmbH) has a bulk density of $0.45\frac{g}{{\mathrm{cm}}^{3}}$ or a relative density of 0.45. The relative tapped density of this material is 0.54. For the considered relative densities in Figure 8, values of commercial powders were chosen. Additionally the mean intensity $I_{m}$ along the width is plotted over the powder bed depth for three relative densities with $n_{med}=1.7$. Unlike the plain surface setup, where the maximum of the absorbed radiation is at the interface, the peak intensity is within the powder bed. This is due to the surface morphology of a powder bed with approximated spherical particles. Commercially-available powders have particles that are not exactly spherical. However, they have a low aspect ratio. The rise from zero to the given relative density of the powder bed needs at least one particle diameter. In the presented examples, this length is between 50μm and 100μm, which correlates well with the mean particle size.

**Figure 9:**
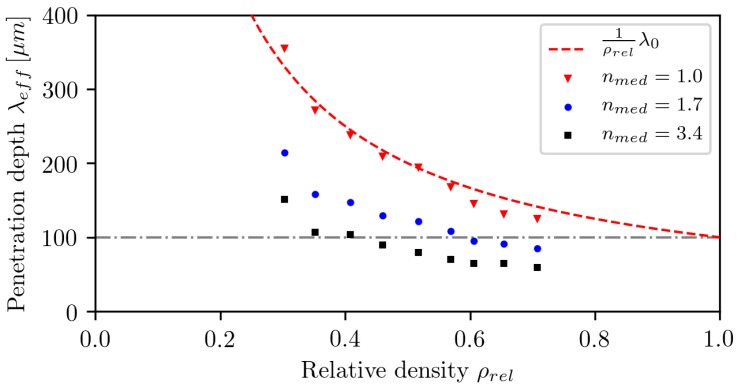
The correlation between $\lambda_{eff}$ and $\rho_{rel}$ for three different refractive indices. The dot-dashed line indicates penetration depth $\lambda_{0}=100\;\mu$m in bulk material.

The effective penetration depth $\lambda_{eff}$ is calculated by fitting Beer–Lambert law (Equation (1)) onto the tail of $I_{m}$ after the peak. The correlation between $\lambda_{eff}$ and $\rho_{rel}$ for three different refractive indices is shown in Figure 9.

For $n_{med}=1$, the effective absorption length is indirectly proportional to the relative density, which is plausible since the rays are not refracted or reflected and therefore propagate along a straight line. On their path, the energy of the ray is only absorbed within the particles, and the fraction of the area covered by dense media is given by $\rho_{rel}$. Therefore, the effective absorption length in this simple case can be calculated by:

(20) $$\begin{array}{c} \lambda_{eff}\left(n_{med}=1\right)=\frac{1}{\rho_{rel}}\lambda_{0} \end{array}$$

For $n_{med}>1$, the rays are refracted and reflected at the interface of particles and the atmosphere. First, the length of the path to reach the same depth is increased by refraction; therefore, more energy is absorbed. Second, the propagation vector of a portion of the reflected rays points towards the surface of the powder bed, and thereby, a net radiation transport is established, increasing the energy fraction absorbed near the surface. These two effects can considerably decrease the effective absorption length. It is shown that this can even decrease the effective penetration depth below the penetration depth in bulk material. It is especially relevant for powders, e.g., PA12, where the penetration depth is larger than the mean particle size $\lambda_{0}>d_{3,50}$, because a significant fraction of the total energy is transmitted beyond the first particle layer, and therefore, more interfaces are crossed. A smaller effective absorption length results in a higher energy density at the powder bed surface and, therefore, in higher peak temperatures during sintering, which can lead to degradation of the polymer. Possible implications need to be considered if using finer particle distributions or increasing the relative powder bed density and thereby increasing the interface density. Furthermore, these effects need to be considered when modeling the process and simplifying the powder to a continuum, since Equation (20) is not sufficient to calculate the penetration depth for $n_{med}\neq 1$.

## 6. Conclusions

An absorption model for the interaction of a light source with a single wavelength (e.g., a laser) in complex geometries, such as a polymeric powder bed, is presented. The model is compared with the analytical solution for a plain surface. Furthermore, the attenuation coefficient for polyamide 12 was measured, and the results were used to simulate the absorptance, reflectance and transmittance in a stochastic powder bed, comparing it with experimental data. Overall, a good agreement was found. Additionally, it was shown that the effective penetration depth can be lower than the penetration depth in bulk material for polymer powders, due to refraction and reflection at the interfaces of particles and the atmosphere. This phenomena increases the energy density absorbed at the surface of the powder bed and can increase the peak temperatures in the top layers. It needs to be considered when modeling the process. Furthermore, it could limit the processability of fine powder distributions at high powder bed densities, due to the increase of peak temperatures.

## References

[1] Schmid M. (2015). Selektives Lasersintern (SLS) mit Kunststoffen: Technologie, Prozesse und Werkstoffe.

[2] Levy G.N., Schindel R., Kruth J. (2003). Rapid manufacturing and rapid tooling with layer manufacturing (LM) technologies, state of the art and future perspectives. CIRP Ann..

[3] Laumer T., Wudy K., Drexler M., Amend P., Roth S., Drummer D., Schmidt M. (2014). Fundamental investigation of laser beam melting of polymers for additive manufacture. J. Laser Appl..

[4] Dupin S., Lame O., Barrès C., Charmeau J.Y. (2012). Microstructural origin of physical and mechanical properties of polyamide 12 processed by laser sintering. Eur. Polym. J..

[5] Drummer D., Drexler M., Kühnlein F. (2012). Effects on the Density Distribution of SLS-Parts. Phys. Procedia.

[6] Foteinopoulos P., Papacharalampopoulos A., Stavropoulos P. (2018). On thermal modeling of Additive Manufacturing processes. CIRP J. Manuf. Sci. Technol..

[7] Riedlbauer D., Drexler M., Drummer D., Steinmann P., Mergheim J. (2014). Modelling, simulation and experimental validation of heat transfer in selective laser melting of the polymeric material PA12. Comput. Mater. Sci..

[8] Kwan A., Dudley J., Lantz E. (2002). Who really discovered Snell’s law?. Phys. World.

[9] Klassen A., Forster V.E., Juechter V., Körner C. (2017). Numerical simulation of multi-component evaporation during selective electron beam melting of TiAl. J. Mater. Process. Technol..

[10] Markl M., Körner C. (2016). Multiscale Modeling of Powder Bed-Based Additive Manufacturing. Annu. Rev. Mater. Res..

[11] Bauereiß A., Scharowsky T., Körner C. (2014). Defect generation and propagation mechanism during additive manufacturing by selective beam melting. J. Mater. Process. Technol..

[12] Thies M. (2005). Lattice Boltzmann Modeling with Free Surfaces Applied to Formation of Metal Foams. Ph.D. Thesis.

[13] Kaddar W. (2010). Die Generative Fertigung Mittels Laser-Sintern: Scanstrategien, Einflüsse Verschiedener Prozessparameter Auf Die Mechanischen und Optischen Eigenschaften Beim LS von Thermoplasten und Deren Nachbearbeitungsmöglichkeiten. Ph.D. Thesis.

[14] Goodridge R., Tuck C., Hague R. (2012). Laser sintering of polyamides and other polymers. Prog. Mater. Sci..

[15] Laumer T., Stichel T., Nagulin K., Schmidt M. (2016). Optical analysis of polymer powder materials for Selective Laser Sintering. Polym. Test..

[16] Drummer D., Drexler M., Wudy K. (2015). Density of Laser Molten Polymer Parts as Function of Powder Coating Process during Additive Manufacturing. Procedia Eng..

